# The Outer Membrane Vesicles of *Salmonella enterica* Serovar Typhimurium Activate Chicken Immune Cells through Lipopolysaccharides and Membrane Proteins

**DOI:** 10.3390/pathogens11030339

**Published:** 2022-03-11

**Authors:** Hongxiao Cui, Yajun Sun, Hua Lin, Yan Zhao, Xin Zhao

**Affiliations:** 1 College of Animal Science and Technology, Northwest A&F University, Yangling, Xianyang 712100, China; cuihx@nwafu.edu.cn (H.C.); Yajun.sun@nwafu.edu.cn (Y.S.); 17208046@nwsuaf.edu.cn (H.L.); zyy1001@nwafu.edu.cn (Y.Z.); 2 Department of Animal Science, McGill University, Montreal, QC H9X 3V9, Canada

**Keywords:** bone marrow-derived monocytes, HD11, spleen mononuclear cells, OMVs, LPS, *S.* Typhimurium

## Abstract

*Salmonella* is a common pathogen which can secrete outer membrane vesicles (OMVs). However, the effect of OMVs from *Salmonella enterica* Serovar Typhimurium (*S.* Typhimurium) of poultry origin on cells of the chicken innate immune system is not well known. In this study, *S.* Typhimurium OMVs were first isolated from three different poultry strains of *Salmonella*, *Salmonella* CVCC542, SALA, and SALB. In order to investigate the effect of OMVs on the maturation of monocytes into macrophages, both bone marrow-derived (BMD) monocytes and macrophage cell line HD11 cells were used. OMVs promoted the formation of monocyte dendrites in both types of cells, enabled BMD cells to become larger, and stimulated expression of LPS-induced TNF-αfactor (LITAF), IL-6, and inducible nitric oxide synthase (iNOS) genes in HD11 cells. These results demonstrated the capability of OMVs to promote the development of chicken monocytes into macrophages and the maturation of macrophages. In order to study the effect of OMVs on the phagocytosis of macrophages, chicken spleen-derived monocytes and HD11 cells were used. Phagocytosis of FITC-*Salmonella* and FITC-dextran by these two types of cells was enhanced after stimulation with OMVs. To determine which components in OMVs were responsible for the above observed results, OMVs were treated with proteinase K(PK) or polymyxin B (PMB). Both treatments reduced the phagocytosis of FITC-*Salmonella* by HD11 cells and chicken spleen mononuclear cells and reduced the secretion of IL-1β, LITAF, and IL-6 cytokines. These results demonstrated that *Salmonella* OMVs activated chicken macrophages and spleen mononuclear cells and the activation was achieved mainly through lipopolysaccharides and membrane proteins.

## 1. Introduction

*Salmonella*-induced foodborne illness is a serious problem for global public health. The major sources of human salmonellosis are contaminated poultry-derived food products, mainly eggs and chicken meat. Using outbreak data up to 2019, the Interagency Food Safety Analytics Collaboration (2021) estimated that around 23.1% of foodborne salmonellosis in the United States was traceable to chickens and eggs [1]. Thus, preventing and reducing *Salmonella* infection in poultry can improve poultry health and reduce salmonellosis in humans. 

Many effective management measures have been developed to control *Salmonella* infection on poultry farms, mainly using sanitary barrier and immune strategies. Among the immune strategies, live-attenuated vaccines, inactivated vaccines, and subunit vaccines have been developed for *Salmonella*, with various degrees of success [2]. In addition to stimulating the immune system to prevent pathogens from causing disease, oral administration of an attenuated *Salmonella enterica* Serovar Typhimurium (*S.* Typhimurium) strain accelerated clearance of *Salmonella* infections through modifications of the gut microbiome [3]. However, the major drawback of live-attenuated *Salmonella* vaccines is poor safety, because the live strain may exist for long periods in chickens as well as in their environment. On the other hand, the shortcoming of inactivated vaccines may be their low protective efficacy due to their quick elimination by the host and low numbers of antigens. The subunit vaccines have not been widely used because they are usually poorly immunogenic, requiring formulation with appropriate adjuvants [2]. Therefore, more efforts are still needed to develop more efficient vaccines against *Salmonella* in chickens. OMVs possess a series of surface antigens with natural conformation and natural properties (such as immunogenicity, self-adjuvant, and immune cell absorption) and can be attractive vaccines against pathogenic bacteria [4]. OMV-based vaccines are also relatively cheap for isolation compared to manufacturing synthetic molecules [5]. OMVs can be naturally produced by Gram-negative bacteria and used in vaccine development, such as *Salmonella* [6] and *Escherichia coli* [7]. *Neisseria meningitidis* OMV vaccines (“Bexsero”) have been approved by the Food and Drug Administration to prevent group B *Neisseria meningitidis* in the United States [8].

Antigen-presenting cells (APCs), such as dendritic cells (DCs), macrophages, and other mononuclear phagocytes, play an important role in the recognition of antigens carried by vaccines. The immune protection of *Salmonella* OMVs as a vaccine has been mainly studied in mice or mouse cell lines. The study of Alaniz et al. [6] has shown that *Salmonella* OMVs as a vaccine activated macrophages and dendritic cells in mice. *Salmonella* OMVs efficiently induced DC maturation (indicated by increased MHC-II and CD86) and robust proinflammatory cytokine production (TNF-α and IL-12) [6]. *Salmonella* OMVs also stimulated mouse J774 macrophages to produce TNF-α and NO, two important inflammatory mediators produced by activated macrophages [6]. *Salmonella* OMVs could be internalized by mouse macrophages RAW264.7 [9].

Chickens are oviparous vertebrates, and their immune system has several major differences from the mammalian immune system, including different Toll-like receptor (TLR) compositions [10] and a unique chicken major histocompatibility complex (MHC) which is roughly 20-fold smaller than the human MHC [11]. TLR receptors and MHC play an important role in antigen recognition and transmission of antigen information. However, whether *Salmonella* OMVs can activate chicken-derived antigen-presenting cells, such as macrophages and monocytes, remains to be studied. 

Monocytes are a type of circulating white blood cell in the blood that play a vital role in host immune defenses and microbial pathogen elimination [12]. Monocytes can mature into macrophages under conditions of inflammation and injury [13]. In the process of inflammation-induced maturation, in addition to changes in surface markers, cell diameter increases (the diameter of circulating monocytes is approximately 7–8 μm, monocyte-derived macrophages 15–20 μm), and increased cytoplasmic complexity (changes in endoplasmic reticulum, mitochondria and lysosomes) [13]. Exposure to granulocyte–macrophage colony-stimulating factor (GM-CSF) and IL-4 induces differentiation of chicken monocytes into dendritic cells [14], whereas exposure to macrophage colony-stimulating factor (M-CSF) induces monocytes to differentiate into chicken macrophages [15]. The initial inflammatory response of the host after *Salmonella* infection may promote the differentiation of macrophages into two main types, the classically activated macrophages (CAMs or M1 type) or the alternatively activated macrophages (AAMs or M2 type) [16]. Studies have found that *Salmonella* prefers inhabiting the M2 type macrophages during the establishment of chronic infections in mice [17]. After differentiation into macrophages, phagocytosis by macrophages is critical for the uptake and degradation of intracellular pathogens and the initiation of the innate immune response [18].

Whether *Salmonella* OMVs could promote differentiation and subsequent phagocytosis by chicken monocytes was examined in this study. In addition, the involvement of lipopolysaccharides (LPS) and proteins in *Salmonella* OMVs in the differentiation of chicken BMD monocytes into chicken macrophages and subsequent phagocytic function was investigated. Our data indicate that the OMVs of *Salmonella* activated chicken macrophages and spleen mononuclear cells mainly through LPS and membrane proteins. 

## 2. Results

### 2.1. The Characterization of Salmonella OMVs

**Figure 1:**
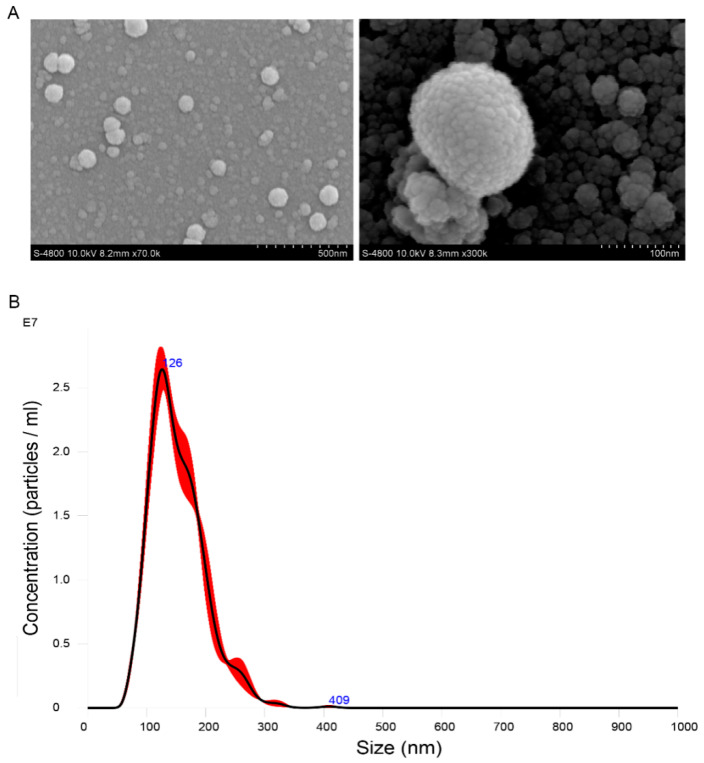
Visualization and characterization of OMVs derived from *Salmonella* CVCC542. (**A**) The scanning electron microscopy (SEM) image of OMVs from *Salmonella*. Bars: 500 nm for the left picture, 100 nm for the right picture. (**B**) The particle number of the isolation layers of OMVs as determined by NanoSight NS300.

To determine the shape and size of OMVs from *S.* Typhimurium CVCC542, SEM and NanoSight NS300 were used. SEM showed that OMVs were spherical particles of diverse sizes, with diameters about 100 nm (Figure 1A). NanoSight NS300 showed the diameters of the OMVs to be in the range of 50–300 nm, with a peak of about 126 nm (Figure 1B). We also determined the concentrations of the protein and the LPS carried by OMVs. As shown in Table 1, the OMVs from strains CVCC542, SALA, and SALB, respectively, contain 0.78 μg, 0.71 μg, and 0.73 μg LPS per μg protein. There were no differences among three different bacteria. Given that their protein and LPS concentrations were not significantly different (Table 1), OMVs of CVCC542 were selected for subsequent experiments.

### 2.2. OMVs Stimulated Formation of Dendrites in Chicken Bone Marrow-Derived Monocytes (BMDMs) and HD11 Cells

**Figure 2:**
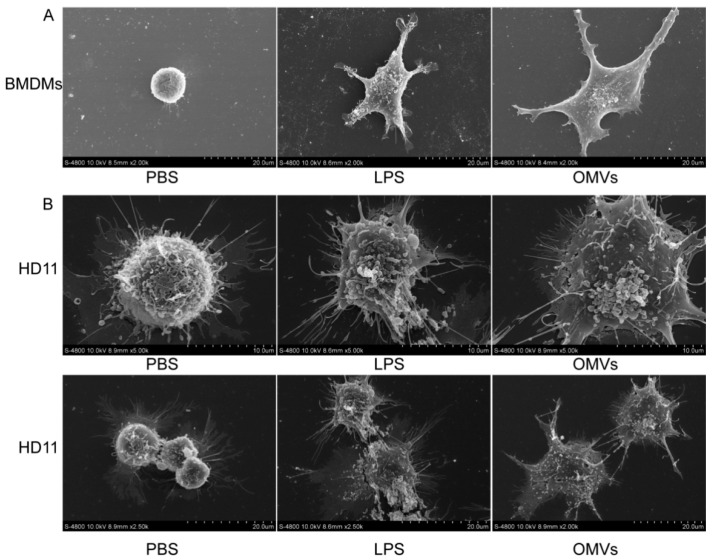
Activation of chicken bone marrow-derived monocytes (**A**) and chicken HD11 cells (**B**). The cells were imaged with an S-4800 SEM. Bars, 20 μm for (**A**), 10 μm and 20 μm for (**B**). BMD monocytes and HD11(10^6^ cells/mL) were incubated with PBS (control), LPS (200 ng/mL; from *Salmonella* enterica serotype typhimurium), and OMVs containing 200 ng/mL LPS for 24 h before SEM observation.

The effect of OMVs on the immune stimulation of bone marrow-derived monocytes from chickens was determined. As shown in Figure 2A, both LPS (a positive control) and OMVs promoted the dendritic formation and increased cellular volume, in comparison with the negative control PBS. 

To confirm the effect of OMVs from *Salmonella* on the activation of antigen-presenting cells, chicken HD11 cells, a macrophage-like immortalized cell line derived from the bone marrow of chickens and transformed with the MC29 virus, were also used. As shown in Figure 2B, the OMVs promoted the formation of HD11 dendrites. Our results indicate that OMVs promoted the dendrite formation of both bone marrow-derived monocytes and HD11 cells. 

### 2.3. OMVs Modulate Cytokine Production in HD11 Cells

**Figure 3:**
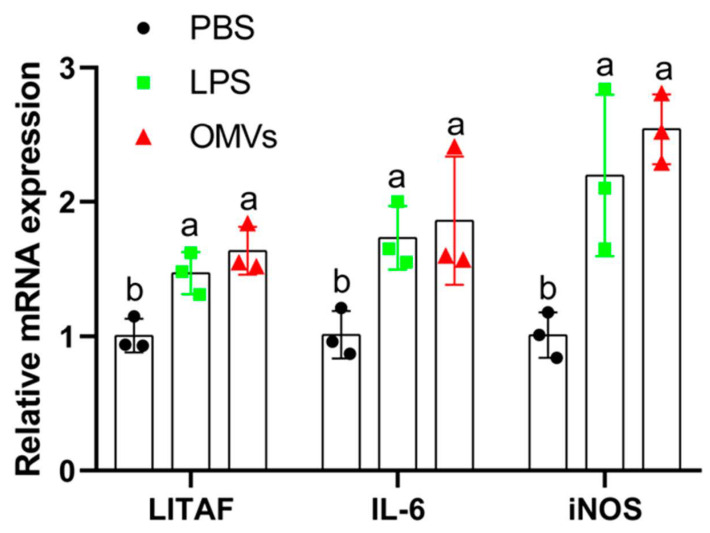
Expression of chicken LITAF, IL6, and iNOS mRNA in chicken HD11 cells. Data are shown as mean ± SD (the standard deviation) from three replicates. Different letters above bars indicate significant differences in relative mRNA expression among different treatments (*p* ≤ 0.05). HD11 (10^6^ cells/mL) were incubated with PBS (control), LPS (200 ng/mL; Sigma-Aldrich, from *Salmonella* enterica serotype typhimurium), and OMVs containing 200 ng/mL LPS for 24 h before extraction of RNAs for gene expression of LITAF, IL-6, and iNOS.

In order to study the activation of antigen-presenting cells by OMVs, mRNA expression of LITAF, IL-6, and iNOS genes in HD11 cells treated with *Salmonella* OMVs was determined. As shown in Figure 3, OMVs significantly increased the mRNA expression of LITAF, IL-6, and iNOS compared with the PBS group, further indicating that *Salmonella* OMVs activated the maturation of macrophage HD11 cells into macrophages.

### 2.4. OMVs Are Internalized by HD11 Cells

**Figure 4:**
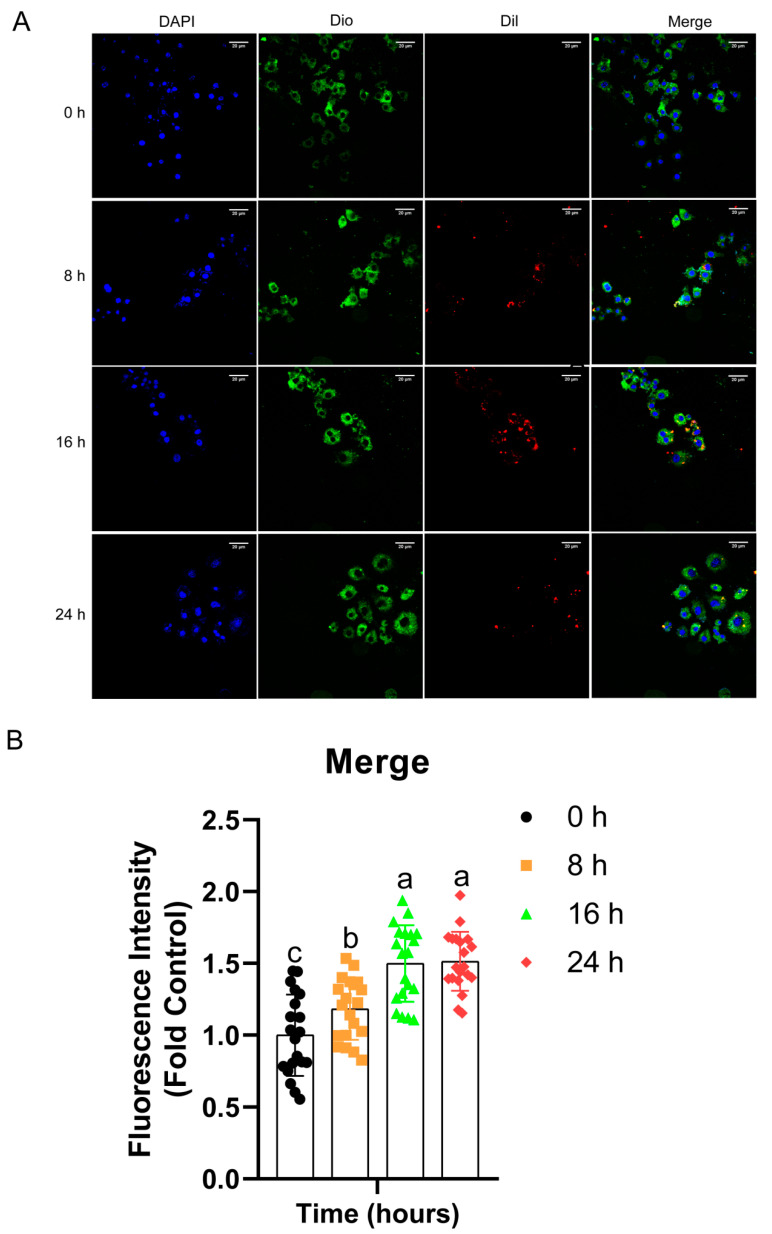
*Salmonella* OMVs uptake by HD11 macrophages cells. DiI-labeled *Salmonella* OMVs containing 200 ng/mL LPS (red signal) were incubated with HD11 cells for 0 h, 8 h, 16 h, and 24 h at 37 ℃. The cell nuclei were stained with DAPI (blue signal) and the membrane of HD11 cells was stained with DIO (green color). The samples were observed using a high-speed spinning-disk confocal microscope (**A**). Bars: 20 μm. The fluorescence intensity of merge cells was determined using Fiji software (**B**). Cells (at 0 h) were used as a control. Data are shown as mean ± SD from twenty replicates. Different letters above bars indicate significant differences of the relative fluorescence intensity (fold control) among different treatments (*p* ≤ 0.05).

To study whether *Salmonella* OMVs could be taken up by HD11 cells, purified OMVs were labeled with DiI (red color), the membrane of HD11 cells was stained with DIO (green color), and the nucleus were stained with DAPI (blue color). The vesicles and cells were co-cultured for 0 h, 8 h, 16 h, and 24 h. As shown in Figure 4, there were red dots within the cells at 8 h, 16 h, and 24 h, suggesting the internalization of OMVs by HD11 cells. 

### 2.5. Salmonella OMVs Improve Phagocytic Capacities of HD11 Cells

**Figure 5:**
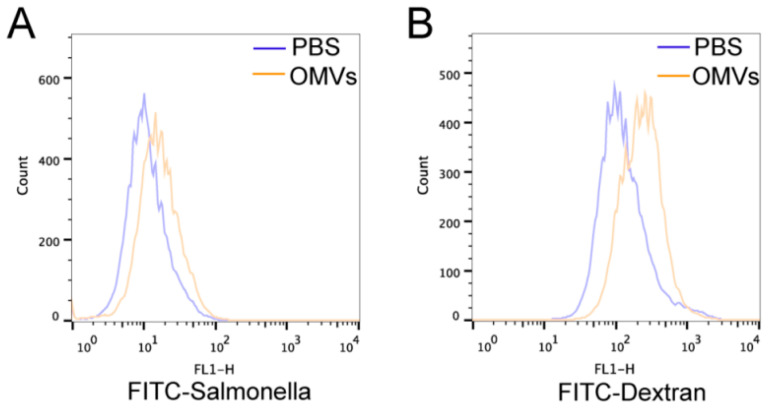
Phagocytosis of FITC-*Salmonella* (**A**) and FITC-dextran (**B**) of HD11 cells stimulated by PBS and OMVs containing 200 ng/mL LPS.

To determine whether *Salmonella* OMVs could increase phagocytic activities, HD11 cells were first cultured for 24 h in the presence or absence of *Salmonella* OMVs and then treated with FITC-dextran or FITC-*Salmonella* for 3 h before detecting the fluorescence intensity and counting CFU numbers of intracellular bacteria. As shown in Figure 5 and Table 2, OMVs significantly increased the fluorescence intensity of the cells compared to the control PBS group. The numbers of CFUs within HD 11 cells were also significantly higher in the OMV-treated cells than in the control group (Figure 5 and Table 2). These results indicated that OMVs enhanced the phagocytic capacities of HD11 cells. 

### 2.6. Involvement of LPS and Proteins from OMVs in the Activation and Phagocytic Capability of the HD11 Cells and Chicken Splenic Mononuclear Cells

*Salmonella* OMVs are rich in LPS and proteins (Table 1). To study the effect of LPS and proteins from OMVs on HD11 cells, purified OMVs were treated with polymyxin B (OMVs + PMB) and proteinase K (OMVs + PK) to neutralize the contribution of LPS and degrade proteins on OMVs, respectively. 

**Figure 6:**
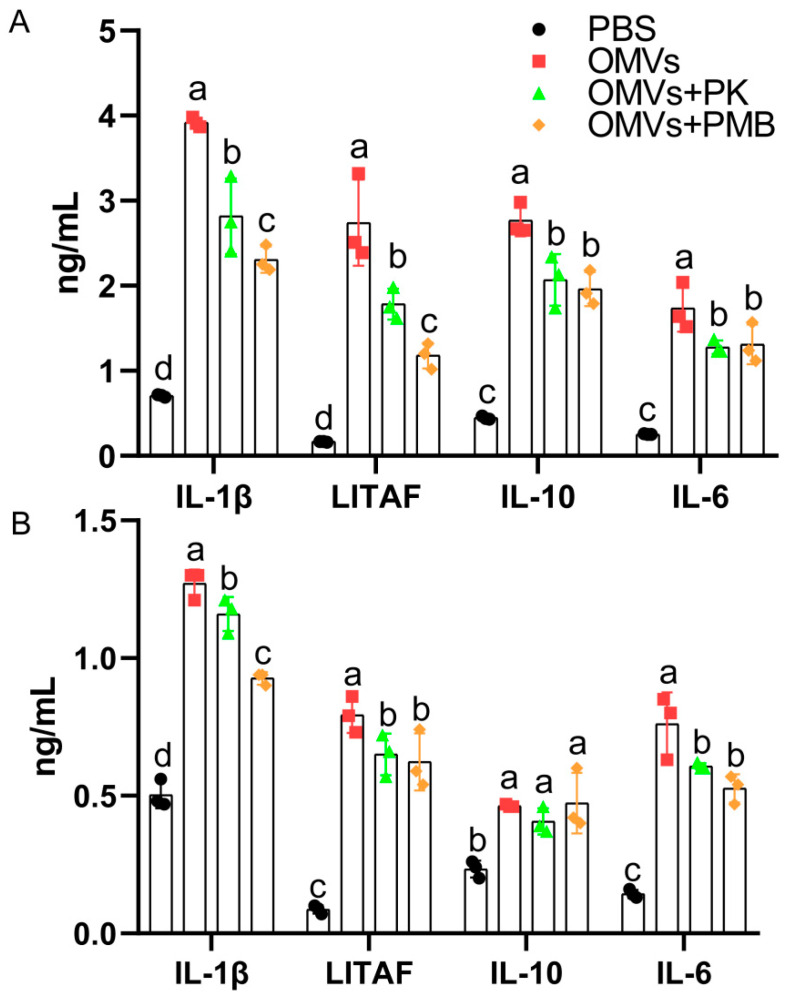
Production of IL-1β, LITAF, IL-10, and IL-6 from the supernatant of HD11 cells (**A**) or chicken splenic mononuclear cells (**B**) after stimulation by OMVs treated with proteinase K or polymyxin B for 24 h. Data are shown as mean ± SD from three independent biological replicates. Different letters above bars indicate significant differences in inflammatory factor concentrations among different treatments (*p* ≤ 0.05).

Cytokines (IL-1β, LITAF, IL-10, and IL-6) in the culture supernatants were quantified after the HD11 cells were incubated with PBS, OMVs, OMVs + PMB, and OMVs + PK for 24 h. As seen in Figure 6A, compared with the OMVs group, secretion of these inflammatory factors was significantly reduced in the OMVs + PMB and OMVs + PK groups. Compared with the OMVs + PK group, the OMVs + PMB group significantly reduced the secretion of IL-1β and LITAF in the cell supernatant. These results indicated that reduction of the proteins or LPS in OMVs affected the immunomodulatory effect of OMVs on HD11, especially after inhibiting LPS.

In order to confirm the results from HD11 cells, we also used chicken splenic mononuclear cells. The chicken spleen mononuclear cells (10^6^ cells/mL) were cultured in 6-well plates and stimulated with PBS, OMVs, OMVs + PMB, and OMVs + PK for 24 h, and then the LITAF, IL-1β, IL-6, and IL-10 levels in the cell supernatant were measured by the ELISA method. As seen in Figure 6B, compared with the OMVs group, the OMVs + PMB and OMVs + PK groups significantly reduced the secretion of LITAF, IL-1β, and IL-6 cytokines. Compared with the OMVs + PK group, the OMVs + PMB group significantly reduced the secretion of IL-1β cytokines, while there was no difference in the secretion of LITAF, IL-10, and IL-6 cytokines between the two groups. These results indicated that proteins or LPS in OMVs had immunomodulatory effects on chicken splenic mononuclear cells. 

**Figure 7:**
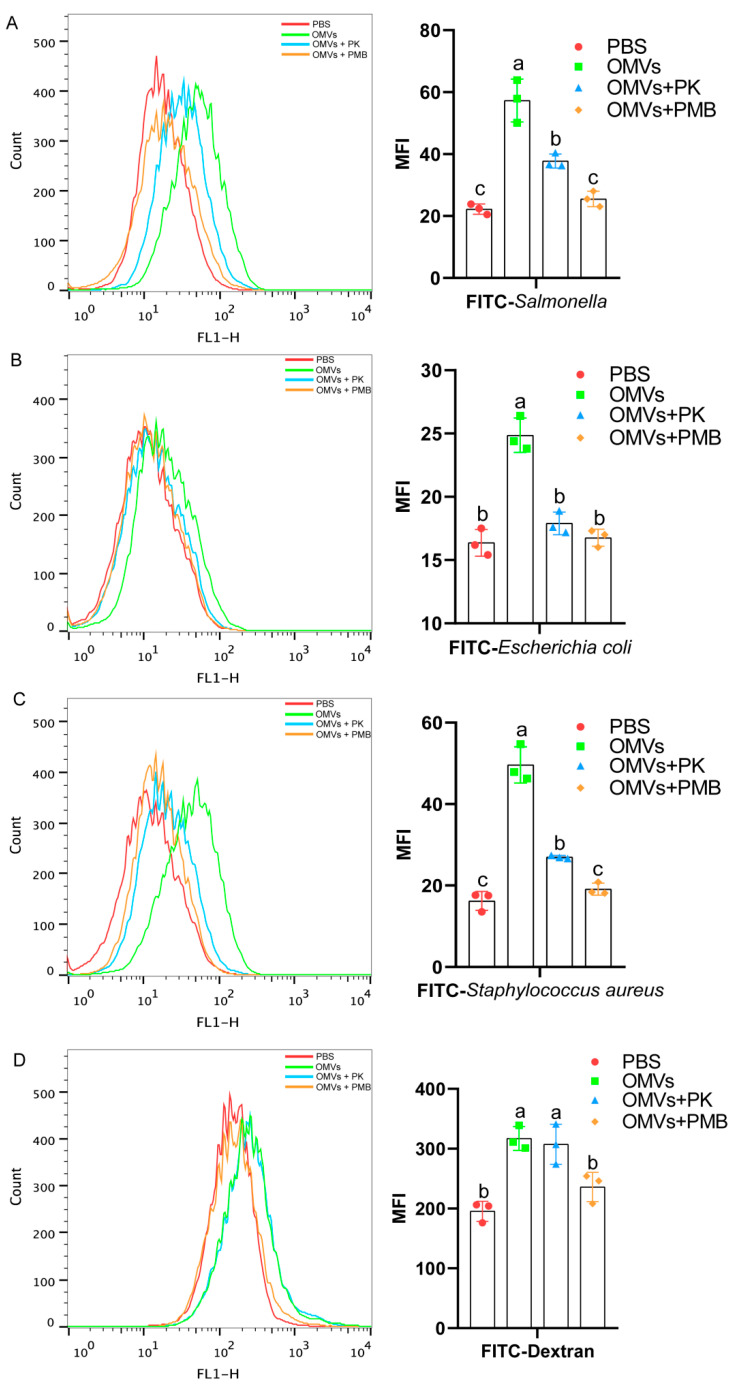
Phagocytosis of FITC-*Salmonella* (**A**), FITC-*E. coli* (**B**), FITC-*Newman* (**C**), and FITC-dextran (**D**) of HD11 cells stimulated by OMVs treated with proteinase K or polymyxin B. HD11 cells were treated with FITC-dextran (1 mg/mL, MW: 40 kDa) or cultured in the presence of FITC-*S.* Typhimurium CVCC542, FITC-*E. coli* and FITC-Newman (multiplicity of infection MOI bacteria/macrophage = 50:1) for three hours. After incubation, the mean fluorescence intensity (MFI) of cells was determined using a flow cytometer. Data are shown as mean ± SD from three independent biological replicates. Different letters above bars indicate significant differences in MFI among different treatments (*p* ≤ 0.05).

To study the effect of LPS and proteins from OMVs on the phagocytic activities of HD11 cells, purified OMVs were treated with polymyxin B (OMVs + PMB) and proteinase K (OMVs + PK) before incubation with HD11 for 24 h. As shown in Figure 7, compared with the OMVs group, both the OMVs + PMB and OMVs + PK groups significantly reduced the phagocytic activities of HD11 for *Salmonella*, *E. Coli* K88, and *Staphylococcus aureus* Newman (Figure 7A–C). Compared with the OMVs + PK group, the OMVs + PMB group significantly reduced the phagocytic activity for FITC-*Salmonella* (Figure 7A), FITC-*Staphylococcus aureus* (Figure 7C), and FITC-dextran (Figure 7D) in HD11. These results indicated that both LPS and proteins in OMVs could enhance the phagocytic activities of chicken macrophages HD11. 

**Figure 8:**
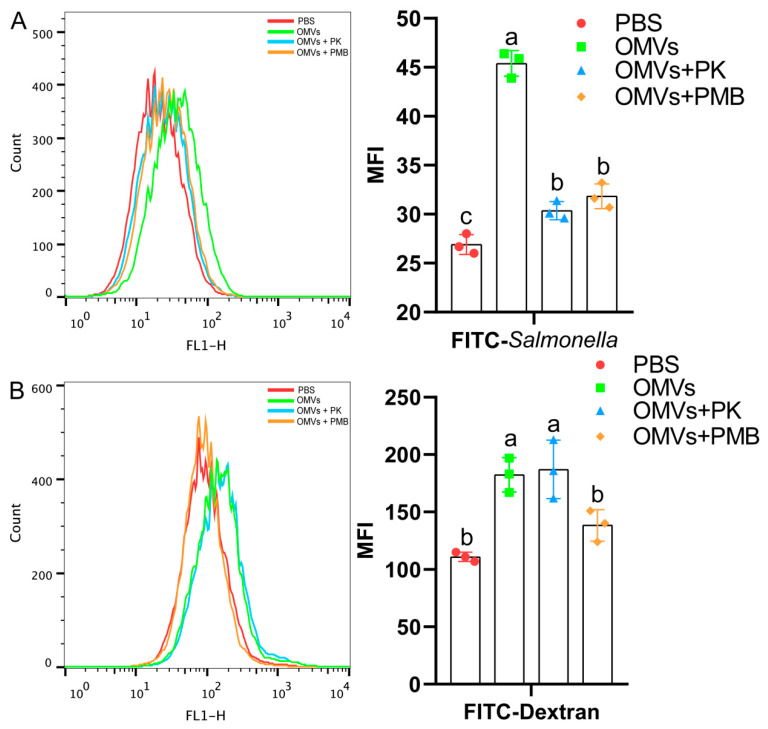
Phagocytosis of FITC-*Salmonella* (**A**) and FITC-dextran (**B**) of chicken splenic mononuclear cells stimulated by OMVs treated with proteinase K or polymyxin B. Chicken splenic mononuclear cells were treated with FITC-dextran (1 mg/mL, MW: 40 kDa) or cultured in the presence of FITC-*S.* Typhimurium CVCC542 (multiplicity of infection MOI bacteria/macrophage = 50:1) for three hours. After incubation, the mean fluorescence intensity (MFI) of cells was determined using a flow cytometer. Data are shown as mean ± SD from three independent biological replicates. Different letters above bars indicate significant differences in MFI among different treatments (*p* ≤ 0.05).

To confirm the effect of LPS and proteins from OMVs on the phagocytic activities in HD11 cells, chicken spleen mononuclear cells were used. Compared with the OMVs group, both the OMVs + PMB and OMVs + PK groups significantly reduced the phagocytosis of FITC-*Salmonella* (Figure 8A), while the OMVs + PMB group significantly reduced the phagocytosis of FITC-dextran by chicken splenic mononuclear cells (Figure 8B). These results demonstrated that LPS and proteins in OMVs enhance the phagocytic activity of chicken splenic mononuclear cells. 

## 3. Discussion

The purpose of the present study was to investigate whether *Salmonella* OMVs as a vaccine could activate chicken-derived antigen-presenting cells such as macrophages and monocytes, and to determine the effect of LPS and proteins on *Salmonella* OMVs on the maturation and subsequent phagocytosis of chicken macrophages and monocytes. 

A good vaccine should have following characteristics: safety [19], accessibility [20], stability (quality-controllable) [21], efficacy [19], and immunogenicity [22]. According to these parameters, our results have demonstrated that *Salmonella* OMVs could be good candidates for a vaccine.

Our study showed that *Salmonella* OMVs are non-replicative. The isolated *Salmonella* OMVs were cultured on the LB board, and no colonies were found (data not shown). *Salmonella* OMVs could be obtained by centrifugation, which is a cheaper isolation method compared to manufacturing synthetic molecules [5]. A previous study found that OMVs were highly stable when exposed to different temperatures and treatments [23]. *Salmonella* OMVs in our study maintained a stable spherical structure after being stored in the refrigerator at −80 degrees Celsius (Figure 1A). Therefore, *Salmonella* OMVs are safe owing to their non-replication and are accessible and stable to be used as a vaccine. 

The size of OMVs is suitable for uptake by antigen-presenting cells. A previous study found that particle sizes of 20–200 nm allow the most efficient uptake by antigen-presenting cells (APCs) and entry into initial lymphatic vessels [24]. In our study, the *Salmonella* OMVs had a nanoparticle size range of 50–300 nm (Figure 1), which promoted the efficient internalization of OMVs by chicken macrophage HD11 cells (Figure 4). Importantly, *Salmonella* OMVs promoted the dendritic formation and increased cellular volume of chicken monocytes cells derived from chicken bone marrow (Figure 2A), in agreement with the results of Kim et al. [25]. OMVs also promoted the dendritic formation of chicken macrophages HD11 (Figure 2B). Therefore, *Salmonella* OMVs could induce the activation and maturation of macrophages and mononuclear phagocytes—one requirement of a potential vaccine candidate. 

HD11 cells activated by *Salmonella* can produce nitric oxide (NO) and secrete a series of cytokines, such as IL-1β, IL-10, and IL-6 [26]. The iNOS genes are involved in the synthesis of NO and could be induced by *Salmonella* [27]. In our study, *Salmonella* OMVs promoted the expression of LITAF, IL-6, and iNOS mRNA in HD11 cells (Figure 3), demonstrating that OMVs from chicken *S.* Typhimurium CVCC542, could promote the activation of chicken antigen-presenting cells HD11, accompanied by the expression of LITAF, IL-6, and iNOS mRNA. Our results were also supported by Mei et al. [28], who observed that the OMVs from the avian bacillus *H. paragallinarum* could stimulate the expression levels of IL-1β, IL-2, IL-6, IL-10, and iNOS in HD11. 

The activation of antigen-presenting cell macrophages could enhance phagocytic activity against microorganisms [29]. Our results showed that *Salmonella* OMVs enhanced the phagocytic function of chicken macrophage HD11 cells (Table 2, Figure 5 and Figure 7) and chicken splenic mononuclear cells (Figure 8). The FITC-*Salmonella* were added according to MOI = 50:1 after staining, while the FITC-dextran was added at 1 mg/mL. The different amounts of fluorescence carried by FITC-*Salmonella* and FITC-dextran may explain the different relative fluorescence intensity observed in the phagocytosis of FITC-*Salmonella* vs. FITC-dextran. Why the phagocytosis of FITC-*Salmonella*, but not FITC-dextran, was reduced after the PK treatment is unknown and needs further investigation. A previous study observed that *E. coli* OMVs enhanced the phagocytic ability of mouse macrophages RAW 264.7 [30]. Oliveira et al. [31] also found that extracellular vesicles from *Cryptococcus neoformans* enhanced the phagocytosis of RAW 264.7 cells. Our results clearly demonstrated that the *Salmonella* OMVs can increase the phagocytosis of bacterial pathogens by chicken macrophages and monocytes, another requirement as a potential vaccine candidate.

OMVs have high immunogenicity and inherent adjuvant effects because they contain many immunogenic components from their parent bacteria, for example, LPS and outer membrane proteins (OMPs) [32]. A previous study found that *Salmonella* OMVs carried LPS and porin (OmpC, OmpF, and OmpD), which could stimulate B cells to produce antibodies, and the presence of the O antigen in LPS acted as an adjuvant leading to increased immunogenicity of OMVs [33]. Here, we found that both LPS and membrane proteins on *Salmonella* OMVs could activate chicken macrophages and spleen mononuclear cells, with LPS playing the main role in OMVs. Similarly, Chu et al. [34] showed that incubating HD11 cells with LPS for 24 h significantly increased the phagocytosis of *Chlamydia psittaci* by macrophages, supporting the notion that LPS is important for the immunostimulatory effect of OMVs. LPS could stimulate the expression of IL-1β, IL-2, IL-6, IL-10, and iNOS in HD11 cells [28]. We also observed that OMVs from *Salmonella* entered the cell (Figure 4), and LPS within the cell can activate inflammasomes and cause the production of IL-1β, as reported by Vanaja et al. [35], who observed that *E. coli* OMVs could carry LPS into the cell to activate the activation of inflammasomes and cause the production of IL-1β. Yang et al. [36] showed that *Salmonella* OMVs directly triggered host NLRC4-mediated canonical inflammasome activation by transporting bacterial flagellin into the cytoplasm of host cells in mice, leading to IL-1β production. In this study, it was found that OMVs also stimulated the secretion of IL-1β, and PK treatment degraded flagellin (data not shown) and reduced the secretion of IL-1β, suggesting that flagellin from *Salmonella* carried as a cargo by the OMVs may play a role in stimulating the secretion of IL-1β inflammatory factor in chicken immune cells. The secretion differences of cytokine IL-10 in HD11 and splenic monocytes may be related to the cells themselves. As an anti-inflammatory factor, IL-10 plays an important role in regulating the balance of inflammation. Our results demonstrated the potential of OMVs from *Salmonella* as a vaccine. This conclusion is supported by a recent report by Li et al. [37], who used recombinant outer membrane protein F (rOmpF) and OMVs from *S.* Enteritidis as a vaccine against *S.* Enteritidis challenge in Hyline White chickens. In return, our study has provided the potential mechanisms explaining why their OMVs vaccine worked.

## 4. Materials and Methods

### 4.1. Bacterial Strains and Chicken Macrophage Cell Line

*S.* Typhimurium CVCC542 was obtained from the China Veterinary Culture Collection Center [38]. SALA and SALB strains were isolated and preserved in our laboratory. We performed 16S rDNA validation on SALA and SALB, as well as *Salmonella* invasive protein gene (InvA) and *Salmonella* Typhimurium specific gene (Sty), on these two isolates. Both SALA and SALB belong to *S.* Typhimurium. *Escherichia coli* F4 (*E. coli* K88) [39] and *Staphylococcus aureus* (*S. aureus*) Newman [40] were preserved in our laboratory. 

CVCC542 and *E. coli* K88 were cultured in Luria–Bertani (LB) medium in a shaking incubator (180 rpm) to OD_600_ = 0.5 at 37 °C. Newman cells were cultured in a tryptic soy broth (TSB) medium in a shaking incubator (180 rpm) to OD_600_ = 0.5 at 37 °C. FITC labeling of *S.* Typhimurium (CVCC542), *S. aureus* (Newman), and *E. coli* K88, was performed using fluorescein isothiocyanate (FITC) staining solution, mainly following the method of Feng et al. [39]. *S.* Typhimurium (CVCC542), *S. aureus* (Newman), and *E. coli* K88 were stained with FITC (10 µg/mL, Sigma, USA) and kept in darkness at 37 °C for 2 h, then washed three times with PBS solution (pH 7.4) to remove the unlabeled FITC and resuspended with PBS at 4 °C. These FITC-labeled bacteria were used in cell phagocytosis assays. The chicken macrophage HD11 cell line was purchased from Otwo Biotech (HTX2259, Shenzhen, China).

### 4.2. Isolation and Characterization of Salmonella OMVs 

The bacterial OMVs were isolated according to a protocol previously described by Prados-Rosales et al. [41] with minor modifications. Briefly, bacteria were cultured in 2 L Luria–Bertani (LB) (1% tryptone, 0.5% yeast extract, 1% NaCl, pH 7.0) in a shaking incubator (180 rpm) to OD_600_ = 1 at 37 °C. After removing bacterial cells by centrifugation at 15,000× *g* for 20 min at 4 °C, the supernatant was filtered through a 0.45 μm filter membrane (Jinteng, Tianjin, China) by a vacuum pump (AP-01 P, Aotu Science, Tianjin, China). The bacteria-free supernatant was concentrated with a 100 kDa membrane (Millipore, Billerica, MA, USA) by the Amicon Ultrafiltration system (Merck Millipore, Billerica, MA, USA). The concentrate was filtered through a 0.22 μm membrane (Millipore, Billerica, MA, USA) to remove any remaining bacteria and was pelleted at 180,000× *g* for 2 h at 4 °C using an ultracentrifuge (Beckman, CA, USA). The pellet containing OMVs was resuspended, washed three times with sterile PBS (pH 7.4), and further purified by OptiPrep (Sigma-Aldrich) density gradient centrifugation (16 h, 200,000× *g*, 4 °C) with Optiprep concentrations ranging from 5% to 45% (*w*/*v*) [42]. 

The fractions containing bacterial OMVs were collected. The concentration of bacterial OMVs from each fraction was measured by a NanoSight NS300 Nanoparticle Tracking analyzer (Malvern Panalytical, Worchestershire, UK). The fractions enriched with OMVs were collected, resuspended in sterile PBS and then centrifuged (2 h, 180,000× *g*, 4 °C) to remove OptiPrep. The purified OMVs were filtered through a 0.22 μm filter to remove debris. Protein concentration was determined with the bicinchoninic acid kit (TaKaRa Bio, Beijing, China). LPS concentration was determined with the LPS kit (Cloud-Clone Corp, Wuhan, China). The OMV sample was stored at −80 °C until use.

Enriched OMVs were analyzed in a NanoSight NS300 nanoparticle analyzer (Malvern Panalytical, Worchestershire, UK) [42]. The diameter sizes and particle numbers of OMVs were monitored. Morphological characteristics of *Salmonella* OMVs were observed by electron microscopy. For scanning electron microscopy (SEM), OMV samples were plated on electron microscope silicon wafers (side length, 4 mm), fixed with glutaraldehyde, washed three times with 0.1 M PBS (pH 7.2), and dehydrated in increasing concentrations of ethanol (30%, 50%, 70%, 80%, 90%, 100%). After that, OMVs samples were plated in isoamyl acetate, critical point dried, coated in gold, and imaged with an S-4800 SEM (Hitachi, Tokyo, Japan) under a beam accelerating voltage of 10 kV. 

### 4.3. Cell Cultures

#### 4.3.1. Generation of Chicken Bone Marrow-Derived Monocyte Cells (BMDMs)

Bone marrow was collected from broiler chickens at four weeks old from a local farm (Youmin Chicken Farm, Wugong, Shaanxi, China). BMDMs were isolated and cultured as previously described [43]. In brief, cells were seeded at 1 × 10^6^ cells/mL in 6-well tissue culture plates in pre-warmed RPMI-1640 (HyClone, Logan, UT, USA), supplemented with 10% fetal bovine serum (Biological Industries BI, Israel), 1% 1 U/mL penicillin and 1 µg/mL streptomycin, 50 ng/mL recombinant chicken granulocyte colony-stimulating factor (GM-CSF, Abcam, Waltham, MA, USA), and 50 ng/mL recombinant chicken IL-4 (Kingfisher Biotech, St Paul, MN, USA) [44]. At day 6, the non-adherent, relatively immature BMD monocytes were harvested and placed in fresh medium (1 × 10^6^ cells/mL) for further experiments. BMD monocytes were incubated with PBS (control), LPS (200 ng/mL; Sigma-Aldrich, from *Salmonella enterica* serotype Typhimurium) and OMVs containing 200 ng/mL LPS for 24 h before SEM observation.

#### 4.3.2. Culture of HD11 Cultures

Chicken HD11 cells were cultured in Roswell Park Memorial Institute (RPMI) 1640 medium (HyClone, USA) supplemented with 10% fetal bovine serum (Biological Industries BI, Israel), 100 U/mL streptomycin, and 100 µg/mL penicillin (Invitrogen, Waltham, MA, USA). Cells in the 6-well plate (Corning, NY, USA) were cultured in a 37 °C incubator with a humidified atmosphere of 5% CO_2_. HD11(10^6^ cells/mL) were incubated with PBS (control), LPS (200 ng/mL; Sigma-Aldrich, from *Salmonella enterica* serotype Typhimurium) and OMVs containing 200 ng/mL LPS for 24 h before SEM observation and extraction of RNAs for gene expression of LITAF, IL-6, and iNOS. RNA was extracted using TRizol reagent (TaKaRa, Dalian, China) based on the manufacturer’s instructions. RNA was reverse-transcribed to cDNA using a Primer Script RT Reagent kit (TaKaRa, Dalian, China). The qRT-PCR was performed using a SYBR Premix Ex Taq kit (TaKaRa, Dalian, China) on a CFX96 Real-Time PCR System (Bio-Rad, USA). The cycle threshold value (CT) was determined, and the relative fold difference was calculated by the 2^−ΔΔCT^ method using GAPDH as the reference gene [45]. The reaction procedures were in accordance with those of Pourabedin et al. [45]. The primers used are listed in Table 3. The primers were synthesized by the Xi’an Qingke Biological Company (Xi’an, Shaanxi, China).

#### 4.3.3. Preparation of Spleen Mononuclear Cells

Spleens were sterilely harvested from four one-day-old Hailan Brown laying chicks (Yangling Julong Poultry Industry Co., Ltd., Yangling, Shaanxi, China). The spleen mononuclear cellsmonocytes were isolated according to a method described by Feng et al. [39]. 

### 4.4. Uptake of Salmonella OMVs by HD11 Cells

To study whether *Salmonella* OMVs could be ingested by HD11 or not, purified OMVs were first labeled with dialkylcarbocyanine iodide DiI (Sigma-Aldrich, St. Louis, MO, USA) fluorescent dye, as described previously [9], bathed in light at 37 °C for 30 min, ultracentrifuged (180,000× *g*, 3 h, 4 °C), and washed with PBS twice, after which the obtained pellet was resuspended in 100 μL of PBS and stored at −80 °C. 

When the HD11 cells in the 6-well plate (Corning, NY, USA) reached 80% confluence, DiI-labeled OMVs containing 200 ng/mL LPS were added to the cells and unlabeled DiI OMVs were used as the control. After further culture for 0, 8, 16, and 24 h, the cells were digested with 0.25% trypsin in a 1.5 mL centrifuge tube for fluorescent staining. After fixation with 4% paraformaldehyde for 30 min in PBS and increasing cell permeability by 0.5% TritonX-100 (Sigma-Aldrich, St. Louis, MO, USA) for 5 min at room temperature, cells were stained with the DIO (1 μM; Beyotime Biotechnology, Shanghai, China) for 15 min at room temperature and counter-stained with DAPI (10 μg/mL; SigmaAldrich, St. Louis, MO, USA) for 15 min at room temperature, then washed with PBS twice. The cells were then placed on polylysine glass slides and observed under a spinning disk confocal microscope (Andor Technology, UK). 

### 4.5. Phagocytic Activities of HD11 Cells 

In order to study whether *Salmonella* OMVs could increase the phagocytic activities of HD11 cells, cells were cultured for 24 h in the presence or absence of *Salmonella* OMVs containing 200 ng/mL LPS. Then, HD11 cells were treated with FITC-dextran (1 mg/mL, MW: 40 kDa, Sigma-Aldrich, St. Louis, MO, USA) or cultured in the presence of FITC-*S.* Typhimurium CVCC542 (multiplicity of infection MOI bacteria/macrophage = 50:1) for three hours. For bacterial treatments, cells were washed 3 times with PBS to remove unattached bacteria. The cells were further incubated with fresh media containing gentamicin (100 μg/mL) [46] before being digested using 0.25% trypsin. One part of the cell lysates was used for the flow cytometer FACS Calibur (BD Biosciences, Franklin Lakes, NJ, USA) to detect the fluorescence intensity, while the other part was lysed with TritonX-100 for plating onto LB agar plates for CFU determination. For each sample, 20,000 cells per sample were recorded using a flow cytometer, and the mean fluorescence intensity (MFI) of each sample was analyzed using the FlowJo software. 

### 4.6. Treatments of OMVs with Polymyxin B and Proteinase K 

Purified OMVs were treated with polymyxin B (OMVs + PMB) and proteinase K (OMVs + PK) using the protocol described previously [47] to neutralize the contribution of LPS and degrade the protein on OMVs, respectively. In brief, purified OMVs were preincubated with 0.1 mg of polymyxin B/mL for 30 min at room temperature prior to the addition of cells. Purified OMVs were treated with 0.1 mg of proteinase K/mL overnight at 37 °C with agitation. Post-incubation, soluble proteins and excess proteinase K were removed from vesicles by three washes in PBS (150,000× *g*, 30 min). PMB can bind LPS and inhibit its functions [48]. We tested the protein concentration of OMVs treated with proteinase K and PMB. The protein concentration of OMVs before the treatment was 1318.14 μg/10^12^ particles. The proteinase K treatment reduced it to 725.17 μg/10^12^ particles, while the PMB treatment did not affect the protein concentration (1254.03 μg/10^12^ particles). Similarly, we measured LPS concentrations of OMVs treated with proteinase K and PMB. While PMB significantly reduced the LPS concentration from 980.24 μg/10^12^ particles to 152.70 μg/10^12^ particles, the proteinase K treatment did not affect the concentration of LPS (980.24 μg/10^12^ particles vs. 889.16 μg/10^12^ particles). Polymyxin B (PMB) is a peptide-based antibiotic that neutralizes LPS by binding with LPS and by modifications of the structure of cholesterol-rich membrane domains and the association of glycosyl phosphatidylinositol (GPI)-anchored proteins [49].

To further study the role of proteins of OMVs in the activation of HD11, HD11 cells (10^6^ cells/mL) were cultured in 6-well plates, and stimulated with PBS, OMVs, OMVs + PMB, and OMVs + PK for 24 h. Cytokines (IL-1β, LITAF, IL-10, and IL-6) in the culture supernatants were quantified using ELISA methods with specific antibodies of LITAF, IL-1β, IL-6, and IL-10 as described previously [39]. The preliminary study indicated that the PMB control group did not affect the phagocytosis and secretion of inflammatory factors of HD11 cells in comparison with the PBS control group. Thus, this group was not considered in subsequent studies.

### 4.7. Statistical Analysis 

Statistical analyses were conducted using SPSS 21.0 (SPSS Inc., Chicago, IL, USA). One-way ANOVA followed by LSD or Dunnett’s T3 multiple comparisons test was performed. Significant differences were defined by *p*-values (two tailed) < 0.05. Different letters (a, b, c, and d) represent significant differences (*p* ≤ 0.05), and the same letters indicate that the two groups are not significant (*p* > 0.05).

## 5. Conclusions

In summary, this study has demonstrated that OMVs from *Salmonella* could activate poultry mononuclear phagocytes and macrophages and that LPS or proteins in OMVs could affect their phagocytic activities and secretion of inflammatory factors. Therefore, this study suggests that OMVs from *Salmonella* could be used as a vaccine candidate for poultry.

## Figures and Tables

**Table 1:** Protein and LPS concentrations in OMVs derived from different *Salmonella* strains.

Bacteria	Proteins (μg/10^12^ Particles)	LPS (μg/10^12^ Particles)	LPS Corresponding to Unit Protein
CVCC542	1342.15 ± 232.10	1038.19 ± 110.45	0.78 ± 0.06
SALA	1365.62 ± 224.33	958.49 ± 221.53	0.71 ± 0.11
SALB	1726.46 ± 587.41	1211.96 ± 200.42	0.73 ± 0.14
*p*-value	0.445	0.318	0.715

Data are shown as mean ± SD (the standard deviation) from three independent biological replicates.

**Table 2:** Mean fluorescence intensity (MFI) of HD11 cells after phagocytosis of FITC-*Salmonella* and FITC-dextran and colony-forming units (CFUs) of intracellular bacteria.

Item	PBS	OMVs
FITC-*Salmonella* (MFI)	13.37 ^b^	20.63 ^a^
	±0.46	±0.59
FITC-dextran (MFI)	179.33 ^b^	275 ^a^
	±10.02	±17.58
Colony-forming unit (10^4^ CFU/mL)	50 ^b^	98.67 ^a^
	±17	±15.14

Data are shown as mean ± SD (the standard deviation) from three independent biological replicates. Different letters (a and b) on the same row indicate significant differences in MFI or CFU among different treatments (*p* ≤ 0.05).

**Table 3:** Primer sequences of genes for real-time PCR.

Gene	Sequence (5′−3′)	Product Size (bp)	GenBank Accession No.
LITAF [45]	F: TACCCTGTCCCACAACCTG	152	XM_015294125.2
	R: TGAACTGGGCGGTCATAGA		
IL-6 [45]	F: ATCCCTCCTCGCCAATCT	142	NM_204628.1
	R: GGCACTGAAACTCCTGGTCT		
iNOS (this study)	F: ATTGTGGAAGGACCGAGCTG	141	NM_204961.1
	R: CCTCGCACACGGTACTCATT		
GAPDH [45]	F: TGGAGAAACCAGCCAAGTAT	145	NM_204305.1
	R: GCATCAAAGGTGGAGGAAT		

## Data Availability

The datasets analyzed in the present study are available from the corresponding author on reasonable request.
